# Acceptability of smartphone text- and voice-based ecological momentary assessment (EMA) methods among low income housing residents in New York City

**DOI:** 10.1186/s13104-017-2850-z

**Published:** 2017-10-26

**Authors:** Dustin T. Duncan, William C. Goedel, James H. Williams, Brian Elbel

**Affiliations:** 1 Department of Population Health, New York University School of Medicine, New York, NY USA; 2 Robert F. Wagner Graduate School of Public Service, New York University, New York, NY USA; 3 Spatial Epidemiology Lab, Department of Population Health, New York University School of Medicine, 227 East 30th Street, Room 621, New York, NY 10016 USA

**Keywords:** Ecological momentary assessment (EMA), Acceptability, Low income populations, Public housing residents, Health disparities

## Abstract

**Objectives:**

This study aimed to evaluate the acceptability of smartphone-based text message- and voice-based ecological momentary assessment (EMA) methods among a sample of low-income housing residents in New York City. Using data from the community-based NYC Low Income Housing, Neighborhoods and Health Study (*n* = 112), the acceptability of text message- and voice-based EMA methods were assessed via survey.

**Results:**

Overall, 88.4% of participants reported that they would participate in a study that utilized text message-based EMA. These analyses showed no appreciable differences by sub-groups (*p* > .05). Overall, 80.2% of participants reported that they would participate in a study that used voice-based EMA. This voice-based method was least acceptable among participants younger than 25 years old compared to participants of all other ages, χ^2^(2) = 10.107, *p* = .006 (among the younger participants 60.7% reported “yes” regarding the anticipated acceptability of voice-based EMA and 39.3% reported “no”). Overall, this work suggests that text message- and voice-based EMA methods are acceptable for use among low-income housing residents. However, the association between age and the acceptability of voice-based EMA suggests that these methods may be less suited for younger populations.

## Introduction

Ecological momentary assessment (EMA) includes a range of methods aimed at capturing data on health behaviors and outcomes in real-time from participants as they experience their daily lives [1]. EMA methods were developed in part in response to the limitations of retrospective recall, as self-reported recall data can frequently be unreliable and are often systematically biased [2]. EMA methods recognize that many behaviors and experiences can be affected by context, so data must be collected on a given experience or behavior in its real-life settings for it to be representative [3]. Studies utilizing EMA often involve many repeated measures over varying durations, affording the temporal resolution needed to assess the dynamics of within-subject changes in behavior and experience over time and across context [4].

These methods have employed various modalities to collect data on behavior in real-time in real world environments, including traditional paper and pencil diaries [5], palm-top computers [6], telephones [7], smartphone applications [8], and collection of medication compliance data by instrumented pill bottles [9]. EMA methods have been employed in a variety of populations to study a variety of behaviors and contexts, including mood and affect [10], tobacco, alcohol, and drug use [6, 11, 12], physical activity and sedentary behavior [13], and eating behaviors [14]. With few exceptions [15–17], most studies utilizing these approaches have been conducted among advantaged populations. As an example of an exception, one EMA study was conducted among a sample of Black men who have sex with men [15]. To our knowledge, no studies have assessed the acceptability of these methods among low-income and racial/ethnic minority populations in an intersectional manner.

Low-income populations are disproportionately impacted by adverse health outcomes, including obesity, diabetes, and hypertension [18]. These health outcomes are affected at the individual-level by diet and exercise, which are in turn, can be affected by the characteristics of the built and food environment at the structural level [19]. Given the limitations of self-reported measures utilized in previous studies of neighborhood contexts and cardiovascular health behaviors in this population [20–22], a more nuanced understanding of these behaviors and contexts may be gained by employing EMA methods. However, previous research has noted that low-income adults are less likely to participate in research studies and community programs [23, 24], underscoring the need to assess the acceptability and feasibility of various EMA methods prior to implementing them. As such, this study aims to evaluate the acceptability of text message- and voice-based EMA methods among a sample of low-income housing residents in New York City (NYC) who were predominantly racial/ethnic minorities. This study additionally aims to assess differences in the acceptability of these methods by demographic sub-groups (e.g., race/ethnicity, education level).

## Main text

### Sample recruitment

Data come from the NYC Low Income Housing, Neighborhoods, and Health Study, a community-based study of neighborhood environments and cardiovascular health among low income housing residents, which has been described elsewhere in detail [25, 26]. Briefly, the overall study included 120 low-income residents, most of whom reported living in public housing. This was a convenience sample, as participants were recruited through community-based approaches, which included distributing flyers outside four selected public housing developments in the Manhattan and Queens boroughs of NYC. In addition, we recruited through flyers posted and circulated by community-based organizations that work with low-income individuals, flyers posted in community locations, and through word of mouth. Inclusion criteria included reporting living in low-income (e.g., public) housing in NYC, being 18 years old or older, being able to speak English, self-reporting not being pregnant, self-reporting no restrictions to usual physical activity, and being willing to wear a global positioning system (GPS) device for 1 week.

### Self-administered survey measures

#### Acceptability of smartphone-based ecological momentary assessment (EMA) methods

The acceptability of two different EMA methods were assessed with two items: “Would you participate in a study that sent you texts via a smartphone asking you questions about your current mood, surroundings, and feelings?” (text message-based EMA) and “Would you participate in a study that called you to ask questions about your current mood, surroundings, and feelings?” (voice-based EMA). Response options for these two items were “Yes” and “No.”

#### Cell phone ownership and use

Cell phone use was assessed with one item reading, “Have you previously used a cell phone?” with two response options (yes, no). Cell phone ownership was assessed with one item reading, “Do you have a cell phone?” with two response options (yes, no). If an individual reported cell phone ownership, they were asked, “Do you own a smartphone?” with two response options (yes, no). If an individual reported smartphone ownership, they were asked “What is the operating system?” with four response options (Apple, Android, Blackberry, Other).

### Objective measures

#### Blood pressure and body mass index (BMI)

The blood pressure and BMI protocols have been described in detail elsewhere and were collected at our research office the day the survey was administered [27, 28]. In brief, participant height and weight were measured to the nearest tenth of a centimeter and to the nearest tenth of a kilogram. These measurements were then used to calculate BMI using standard formulas. BMI under 18.5 were classified as underweight, between 18.5 and 24.9 were classified as normal weight, between 25.0 and 29.9 were classified as overweight, and 30.0 and over were classified as obese. Blood pressure was measured a single time in the seated position with the participants’ legs uncrossed and arms outstretched after the participants had been seated for 15–30 s, using a Welch Allyn Vital Signs 300 monitor. Measured hypertension was classified as a systolic pressure ≥ 140 mmHg or a diastolic pressure ≥ 90 mmHg. Pre-hypertension was classified as a systolic pressure between 120 and 139 mmHg or a diastolic pressure between 80 and 89 mmHg. Normal blood pressure was classified as a systolic pressure below 120 mmHg and a diastolic pressure below 80 mmHg [29].

#### Covariates

Participants reported age (years), gender (male, female), race/ethnicity (White, Black, Hispanic, Asian, Other), household income (less than $25,000; $25,000 to $49,999; $50,000 to $74,999; $75,000 or greater), educational attainment (less than 12th grade, high school or GED, some college, bachelor’s degree, graduate degree), employment status (working full-time, working part-time, not working, retired, in school), and health insurance status (yes, no). This information was collected via survey.

### Statistical analyses

The analytical sample was restricted to participants who answered both EMA acceptability items (*n* = 112), representing 94.2% of the overall sample. Descriptive statistics (e.g. frequencies) were calculated for all variables. Differences in acceptability of each of the EMA methods by socio-demographic characteristics and health status were assessed using Chi square tests. Statistical significance was set at *p* < .05.

## Results

### Sample demographics

The demographics of the sample (*n* = 112) are reported in Table 1. The average age of the sample was 38.7 years old (SD = 14.3). More than half (55.4%) of the participants were female. Most participants (71.5%) identified their race/ethnicity as Black/African American or Hispanic/Latino. In addition, most (71.4%) reported an income lower than $25,000. A majority (71.4%) completed high school or some equivalent designation. Few reported working full or part time (34.8%), where 51.8% reported not currently working. Over two-thirds of the sample (70.5%) had a BMI classified as overweight or obese, where 42.9% were obese. Two-thirds (67.0%) had a blood pressure classified as hypertensive or pre-hypertensive, where 31.3% were hypertensive.

**Table 1:** Sample demographics (*n* = 112)

	% (*n*)
Age	
Less than 25 years old	25.0 (28)
25 to 44 years old	34.8 (39)
45 years old and older	40.2 (45)
Gender	
Male	42.9 (48)
Female	55.4 (62)
Race/ethnicity	
White/Caucasian	.9 (1)
Black/African American	67.0 (75)
Hispanic/Latino	4.5 (5)
Asian/Pacific Islander	2.7 (3)
Multiracial/other	23.2 (26)
Educational attainment	
Less than 12th grade	27.7 (31)
High school or equivalent	40.2 (45)
Some college	24.1 (27)
Bachelor’s degree or higher	7.2 (8)
Household income	
Less than $25,000	71.4 (80)
$25,000–$49,999	22.3 (25)
$50,000 or greater	5.4 (6)
Employment status	
Working full time	15.2 (17)
Working part time	19.6 (22)
Not working	51.8 (58)
Retired	4.5 (6)
Student	6.3 (7)
Body mass index classification	
Underweight	1.8 (2)
Normal weight	27.7 (31)
Overweight	27.7 (31)
Obese	42.9 (48)
Blood pressure classification	
Normal blood pressure	29.5 (33)
Pre-hypertension	30.4 (34)
Hypertension	40.2 (45)
Cell phone ownership	
Yes	92.0 (103)
No	8.0 (9)
Smartphone ownership	
Yes	57.4 (58)
No	42.6 (34)

### Cell phone use and ownership

Almost all participants (96.4%) reported previously using a cell phone. Most participants (92.0%) owned a cell phone. Among those who reported cell phone ownership (*n* = 103), 56.3% owned a smartphone. Among those who reported owning smartphone (*n* = 58), 63.8% reported owning Android-based smartphones, 25.9% reported owning Apple-based smartphones, 1.7% reported owning Blackberry-based smartphones, and 8.6% reported owning a smartphone that used another operating system.

### Acceptability of ecological momentary assessment methods

Overall, 88.4% of participants reported that they would participate in a study that sent them text messages via smartphone asking them questions about their current mood, surroundings, and feelings. Differences in acceptability of text message-based EMA methods by socio-demographic characteristics, health status, and cell phone ownership and use are displayed in Table 2. These analyses showed no appreciable differences by sub-groups (*p* > .05).

**Table 2:** Acceptability of text message-based ecological momentary assessment methods

	Yes (% [*n*])	No (% [*n*])	*p* value
Age			.15
Less than 25 years old	78.6 (22)	21.4 (22)	
25 to 44 years old	89.7 (35)	10.3 (4)	
45 years old and older	93.3 (42)	6.7 (3)	
Gender			.63
Male	85.4 (41)	14.6 (7)	
Female	90.3 (56)	9.7 (6)	
Race/ethnicity			.80
White/Caucasian	100.0 (1)	–	
Black/African American	89.3 (67)	10.7 (8)	
Hispanic/Latino	100.0 (5)	–	
Asian/Pacific Islander	100.0 (3)	–	
Multiracial/other	84.6 (22)	15.4 (4)	
Educational attainment			.90
Less than 12th grade	87.1 (27)	12.9 (4)	
High school or equivalent	86.7 (39)	13.3 (6)	
Some college	92.6 (25)	7.4 (2)	
Bachelor’s degree or higher	87.5 (7)	12.5 (1)	
Household income			.84
Less than $25,000	87.5 (70)	12.5 (10)	
$25,000–$49,999	92.0 (23)	8.0 (2)	
$50,000 or greater	83.3 (5)	16.7 (1)	
Employment status			.48
Working full time	94.1 (16)	5.9 (1)	
Working part time	90.9 (20)	9.1 (2)	
Not working	87.9 (51)	12.1 (7)	
Retired	66.7 (4)	33.3 (2)	
Student	85.7 (6)	14.3 (1)	
Body mass index classification			.40
Underweight	100.0 (2)		
Normal weight	80.6 (25)	19.4 (6)	
Overweight	93.5 (29)	6.5 (2)	
Obese	89.6 (43)	10.4 (5)	
Blood pressure classification			.14
Normal blood pressure	81.8 (27)	18.2 (6)	
Pre-hypertension	97.1 (33)	2.9 (1)	
Hypertension	86.7 (39)	13.3 (6)	
Cell phone ownership			.28
Yes	89.3 (92)	10.7 (11)	
No	77.8 (7)	22.2 (2)	
Smartphone ownership			.66
Yes	90.7 (39)	9.3 (4)	
No	87.9 (51)	12.1 (7)	

Overall, 80.2% of participants reported that they would participate in a study that called them to asked them questions about their current mood, surroundings, and feelings. Differences in acceptability of voice-based EMA methods by socio-demographic characteristics, health status, and cell phone ownership and use are shown in Table 3. These methods were less acceptable among individuals younger than 25 years old (60.7%) compared to individuals of other ages, χ^2^(2) = 10.107, *p* = .006 (among the younger participants 60.7% reported “yes” regarding the anticipated acceptability of voice-based EMA and 39.3% reported “no”). Additionally, these methods were less acceptable among individuals who reported currently being in school compared all others, χ^2^(4) = 15.202, *p* = .004 (28.6% versus 71.4%). These were the only observed differences in acceptability and we note that some cells have very small sample sizes.

**Table 3:** Acceptability of voice-based ecological momentary assessment methods

	Yes (% [*n*])	No (% [*n*])	*p* value
Age			.01
Less than 25 years old	60.7 (17)	39.3 (11)	
25 to 44 years old	81.6 (31)	18.4 (7)	
45 years old and older	91.1 (41)	8.9 (4)	
Gender			.09
Male	72.3 (34)	27.7 (13)	
Female	85.5 (53)	14.5 (9)	
Race/ethnicity			.91
White/Caucasian	100.0 (1)	–	
Black/African American	79.7 (59)	20.3 (15)	
Hispanic/Latino	80.0 (4)	20.0 (1)	
Asian/Pacific Islander	100.0 (3)	–	
Multiracial/other	80.8 (21)	19.2 (5)	
Educational attainment			.88
Less than 12th grade	80.6 (25)	19.4 (6)	
High school or equivalent	79.5 (35)	20.5 (9)	
Some college	81.5 (22)	18.5 (5)	
Bachelor’s degree or higher	75.0 (6)	25.0 (2)	
Household income			.26
Less than $25,000	81.0 (64)	19.0 (15)	
$25,000–$49,999	80.0 (20)	20.0 (5)	
$50,000 or greater	66.7 (4)	33.3 (2)	
Employment status			.00
Working full time	94.1 (16)	5.9 (1)	
Working part time	77.3 (17)	22.7 (5)	
Not working	80.7 (46)	19.3 (11)	
Retired	100.0 (6)	–	
Student	28.6 (2)	71.4 (5)	
Body mass index classification			.28
Underweight	50.0 (1)	50.0 (1)	
Normal weight	71.0 (22)	29.0 (9)	
Overweight	83.9 (26)	16.1 (5)	
Obese	85.1 (40)	14.9 (7)	
Blood pressure classification			.43
Normal blood pressure	72.7 (24)	27.3 (24)	
Pre-hypertension	82.4 (28)	17.6 (6)	
Hypertension	84.1 (37)	15.9 (7)	
Cell Phone ownership			.29
Yes	81.4 (83)	18.6 (19)	
No	66.7 (6)	33.3 (3)	
Smartphone ownership			.35
Yes	84.2 (48)	15.8 (9)	
No	76.7 (33)	23.3 (10)	

## Discussion

Overall, this work suggests that smartphone-based text message- and voice-based EMA methods are acceptable for use among low-income housing residents who were predominantly racial/ethnic minorities. Specifically, the lack of significant variations in acceptability of text message-based EMA suggests that these methods can be implemented in diverse populations of low income housing residents regardless of socio-demographic characteristics and cellphone ownership and use, which has tremendous implications for EMA research in this unique population. However, the association between age and the acceptability of voice-based EMA suggests that these methods may be better suited for older populations of low-income housing residents. To our knowledge, this is the first study to examine the acceptability of various EMA methods among low-income housing residents. As discussed previously, overall, the majority of studies using EMA approaches have been conducted among advantaged populations.

Future research would benefit from the utilization of these highly innovative methods among this population to overcome the limitations of traditional survey-based assessments of poor health behaviors, which are highly prevent among low incoming housing residents [20–22, 28, 30]. For example, EMA via palmtop computers was recently used to collect real-time information about participants’ environment and eating patterns to predict overeating that could lead to weight gain among a sample of 39 undergraduate women [31]. EMA methods administered via smartphones have been paired with accelerometer use among a sample of 110 adults to examine physical activity and sedentary behaviors [32]. Beyond addressing traditional limitations of research, EMA methods offer new avenues for engagement and inquiry for collecting data using mobile technologies real-time and tailoring behavioral health interventions to specific spatio-temporal contexts.

## Conclusion

This study provides evidence for the acceptability of EMA methods among low-incoming housing residents. The lack of significant variations in acceptability of text message-based EMA suggests that these methods can be implemented in diverse populations of low-income housing residents regardless of socio-demographic characteristics and cellphone ownership and use. However, the association between age and the acceptability of voice-based EMA suggests that these methods may be less suited for younger populations.

## Limitations

While our study has several important strengths (including a sample of predominantly racial/ethnic minority low-income housing residents), our study also has limitations. First, we note that this was a relatively small convenience sample of low-income housing residents in NYC who perhaps were motivated to engage in research. Consequently, our findings might not be generalizable to all low-income populations, including those in other geographic regions especially those in rural geographies. However, our sample included a diverse sample of low-income adults across different NYC neighborhoods [27]. Also, given that 20% of Americans now live in the 100 largest cities, and more than 70% of Americans living in urban areas with urbanization still on the rise, the relevance is large and growing [33]. Second, our study was limited to English speaking low-income housing residents. As such, our results may not be generalizable to non-English speaking low-income housing populations. In addition, social desirability bias might be a concern. These data are 3 years old, so there may be have been changes overtime in the acceptability of EMA methods (perhaps a wider acceptance).

## Abbreviations

BP: blood pressure
BMI: body mass index
EMA: ecological momentary assessment
NYC: New York City
